# Caesarean Section: Could Different Transverse Abdominal Incision Techniques Influence Postpartum Pain and Subsequent Quality of Life? A Systematic Review

**DOI:** 10.1371/journal.pone.0114190

**Published:** 2015-02-03

**Authors:** Salvatore Gizzo, Alessandra Andrisani, Marco Noventa, Stefania Di Gangi, Michela Quaranta, Erich Cosmi, Donato D’Antona, Giovanni Battista Nardelli, Guido Ambrosini

**Affiliations:** 1 Department of Women’s and Children’s Health—University of Padua, Padua, Italy; 2 Department of Obstetrics and Gynecology, University of Verona, Verona, Italy; Oslo University Hospital, Ullevål, NORWAY

## Abstract

The choice of the type of abdominal incision performed in caesarean delivery is made chiefly on the basis of the individual surgeon’s experience and preference. A general consensus on the most appropriate surgical technique has not yet been reached. The aim of this systematic review of the literature is to compare the two most commonly used transverse abdominal incisions for caesarean delivery, the Pfannenstiel incision and the modified Joel-Cohen incision, in terms of acute and chronic post-surgical pain and their subsequent influence in terms of quality of life. Electronic database searches formed the basis of the literature search and the following databases were searched in the time frame between January 1997 and December 2013: MEDLINE, EMBASE Sciencedirect and the Cochrane Library. Key search terms included: “acute pain”, “chronic pain”, “Pfannenstiel incision”, “Misgav-Ladach”, “Joel Cohen incision”, in combination with “Caesarean Section”, “abdominal incision”, “numbness”, “neuropathic pain” and “nerve entrapment”. Data on 4771 patients who underwent caesarean section (CS) was collected with regards to the relation between surgical techniques and postoperative outcomes defined as acute or chronic pain and future pregnancy desire. The Misgav-Ladach incision was associated with a significant advantage in terms of reduction of post-surgical acute and chronic pain. It was indicated as the optimal technique in view of its characteristic of reducing lower pelvic discomfort and pain, thus improving quality of life and future fertility desire. Further studies which are not subject to important bias like pre-existing chronic pain, non-standardized analgesia administration, variable length of skin incision and previous abdominal surgery are required.

## Introduction

Caesarean section (CS) has become one of the most performed surgical procedures worldwide. Both in Italy and in the United States, CS rates have increased dramatically over the past decades. The rising trend in caesarean delivery from 11.2% in the 80s to 27.9% in 1996 to a soaring 38% in 2009 sees Italy rank first place among European Countries.[1,2]

Several CS skin incision and abdominal wall opening techniques have been developed during the years, yet a general consensus on the most appropriate approach, in terms of safety and morbidity, has not been yet reached. The choice of technique depends largely on the Surgeon’s experience and preference and on the maternal-fetal clinical condition. [3,4]

Acute and chronic pain after CS depends mainly on the type of cutaneous incision and subsequent access into the pelvic cavity, in relation to the abdominal wall’s somatic innervation.[5] The evaluation of post-surgical wellbeing benefits from the use of subjective and objective pain scores in both early and long term follow-up.

Nerve injury or entrapment occurring during or following surgical procedures seems to be the main cause responsible for the occurrence of chronic pain after CS. [6]

The Pfannenstiel incision, also known as the “bikini incision”, and the Misgav-Ladach method, mainly represented by the modified Joel-Cohen incision, are the most common skin incisions performed. [7]

The former is a transverse “smile”-like incision made 2–3 cm above the symphysis pubis at the pubic area border; the latter is a straight transverse skin incision which lies about 3 cm below the level of the anterior superior iliac spines (ASIS). Both techniques involve skin and subcutaneous tissues. Although several studies comparing these two abdominal wall opening techniques have been conducted, differences in terms of acute and chronic post-operative pain have not been always considered. [4,8–14]

The aim of this systematic surgical overview and literature review is to compare the effect of the Pfannenstiel incision as opposed to the modified Joel-Cohen incision performed during caesarean delivery in terms of post-surgical acute and chronic pain, and the subsequent effect on quality of life (QoL).

## Materials and Methods

### Data Collection And Analysis

A systematic Literature analysis through electronic databases MEDLINE, EMBASE Sciencedirect and the Cochrane Library in the period between January 1997 and December 2013 was conducted.

Articles in English and French languages were included.

Regarding the manuscript type, we considered eligible all original descriptions, case series, and retrospective evaluations which compared or described outcomes concerning acute and/or chronic pain after CS.

Key search terms included: “acute pain”, “chronic pain”, “Pfannenstiel incision”, “Misgav-Ladach incision”, “Joel-Cohen incision”, in combination with “Caesarean Section”, “abdominal incision”, “numbness”, “neuropathic pain” and “nerve entrapment”.

A manual search of the reference list of included studies and review articles was subsequently performed. References of retrieved articles were analyzed to identify any which may have been potentially missed in the initial research.

We considered eligibility criteria the availability of data regarding the following: type of CS incision (Pfannenstiel incision and/or Misgav-Ladach/Joel-Cohen incision) with the description of details concerning incision length, acute and chronic pain (VAS scale and timing of onset) type of CS (first or repeat CS, elective or urgent) as well as the standard epidemiological features of the patients involved in the studies. Post-operative pain was considered in relation to onset time from CS performance. Data was divided into five temporal classes (class 1: 24–48 hours, class 2: 1–3 weeks; class 3: 4 weeks-2 months; class 4: 3–6 months; class 5: 1 year and beyond) reporting explicit or extracted data from studies. When not clearly identifiable, we categorized pain in acute or chronic. When the pain scale adopted differed from the VAS, we converted the values into VAS scores according to criteria defined by Breivik et al. [15]

Our endpoint was to evaluate eventual postoperative differences between the Pfannenstiel and/or Misgav-Ladach/Joel-Cohen incision, used for the abdominal wall opening during CS, in terms of acute and chronic abdominal pain. In addition, when possible, we analyzed whether factors other than surgical technique (epidemiological features, obstetrical cofactors) may influence the postoperative pain.

Studies providing ambiguous or insufficient data regarding procedure or following outcomes were excluded.

### Abdominal Innervation

Both techniques involve an abdominal area innervated by two principal nerves: ileo-hypogastric and ileo-inguinal. These nerves originate from the lumbar plexus, which is formed by the ventral branches of the first to the fourth lumbar nerves (L1-L4) and by the last thoracic nerve (T12) supplementing with a twig.[5] The lumbar plexus also gives rise to 4 additional peripheral nerves: genito-femoral, lateral femoral cutaneous, femoral and obturator nerves.

The ileo-hypogastric nerve is formed by the fusion of the first lumbar branch with fibers originating from T12. It arises from the upper part of the lateral border of the psoas major then courses infra-laterally atop the quadratus lumborum to the ilium crest where it pierces the transverse abdominal muscle and emerges approximately 3 cm medial to the ASIS. The proximal end of the ileo-hypogastric nerve enters the abdominal wall 2.8±1.3 cm medial to and 1.4±1.2 cm inferior to the ASIS. Once in the abdominal wall, it follows a linear path terminating 4±1.3 cm lateral to the midline.[5,16] As the ileo-hypogastric passes through the abdominal oblique muscles, it divides into the lateral and anterior cutaneous branches which provide sensory innervation to the gluteal (lateral cutaneous branch) and the hypogastric skin regions (anterior cutaneous branch).

The ileo-inguinal nerve is the inferior branch of the L1 root. It follows the same pathway as the ileo-hypogastric nerve but deviates caudally, closer to inguinal ligament. It enters the abdominal wall at a mean distance of 2.8±1.1 cm medial to and 4±1.2 cm inferior to the ASIS, and, similiarly to its superior branch, then follows a linear path terminating 3±0.5 cm lateral to the midline.[16] It is a mixed nerve containing both sensory (groin and neighbouring regions) and motor fibers (large abdominal muscles).

### Surgical Procedures

The Pfannenstiel is probably the most performed incision in obstetrics and gynecology for the following reasons: it offers adequate pelvic exposure, excellent postoperative strength, and satisfactory cosmetic results. In preoperative assessment, the Surgeon should evaluate the need for organ and tissue exposure beyond the pelvis and capability of reaching the target organ in safety.[7,17]

Skin incision is made transversely about 3 cm above the pubic symphysis at the pubic area border, usually for 10 cm-length (ranging 8 to 12 cm). Subcutaneous fatty tissue and abdominal rectus muscles are separated following sharp incision of the internal oblique, external oblique and transversus muscles fascia. Subsequently, the aponeurosis, including linea alba fibers, is separated from the pyramidalis and rectus muscles and is extended for about 6 cm vertically in a cranial direction [from symphysis to umbilicus]. Muscles are then divided at the midline by separating the transversalis fascia and posterior rectus fascia (cranially respect arcuate line). Perforating blood vessels may be clotted with electro-cautery or clamped and cut, if required, in order to achieve hemostasis. The parietal peritoneum is opened by sharp dissection. Retractor insertion is necessary for optimal exposure of the surgical site. Uterine incision, fetal extraction, and uterine suture are performed according to the well-known technique.[7,17] Parietal peritoneum is closed with a continuous suture (using 2–0 polyglactin suture); rectus muscles with single approximating sutures (using 2–0 polyglactin suture); fascia with a continuous locking suture (using 1–0 polyglactin suture); subcutaneous tissue by interrupted stitches (using 2–0 polyglactin suture). Finally, skin approximation is achievd by intradermal suture (using 4–0 polydioxanone).

The Misgav-Ladach method differs from the Pfannenstiel mainly for the modified Joel-Cohen abdominal approach.[7,18] In the Joel-Cohen method, skin incision is performed about 3 cm below the ASIS line for a variable length of 15–17 cm and deepened at the midline for 3–4 cm till exposure of the anterior rectus sheath. The former is transversely incised bilaterally below the fatty tissue. Blunt dissection of the fascia is performed by insertion of the index finger at the midline of the rectus muscles; the fascia is then stretched in the cranial caudal direction by the surgeon and the assistant. Rectus muscles and parietal peritoneum are held and pulled away from the midline to the corresponding side.[7,18] Following fetal extraction and uterine suture, the parietal peritoneum is left open. The fascia is closed by a continuous non-locking suture (using 1–0 polyglactin suture) and the skin by intradermal suture (using polydioxanone 4–0).

## Results

**Figure 1:**
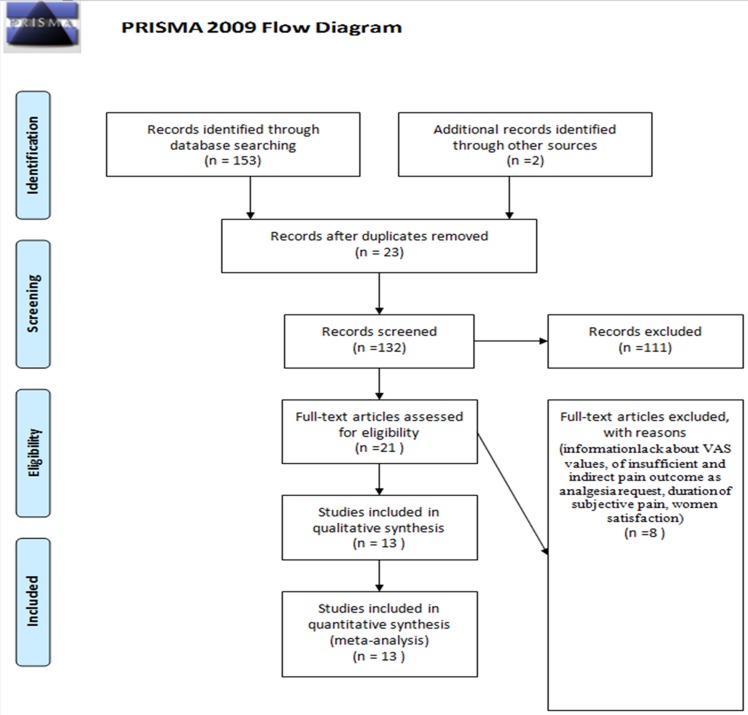
Flow diagram.

More than 150 articles were available in literature for the time period considered however only 21 satisfied our selection criteria. A total of only 13 articles were considered in the analysis [8–12,14,19–25] (Tables 1,2), while the remaining were not included [3,26–32] due to the following: lack of information regarding VAS scores, insufficient data pertaining to direct and indirect pain outcome, type of analgesia requested, duration of subjective pain, and patient satisfaction. (Fig. 1, Table 3)

**Table 1:** Baseline data, study design and surgical features of the reviewed population.

**Author (Year)**	**Type of Study**	**Abdominal opening technique**	**Total scar length (distance above pubis)**	**Number of patients (PF vs ML)**	**Average Age**	**Previous CS (number)**	**Previous Pain (%)**	**Previous abdominal surgery (%)**	**Spinal Anesthesia (%)**	**Urgent CS (%)**
**Hojberg et al (1998)**	1	PF	-	40	31	-	-	35	92	-
**Ferrari et al (2000)**	1	PF vs ML	-	158 (75 vs 83)	31	-	-	-	40 (50 vs 31)	32 vs 45
**Ansaloni et al (2001)**	1	PF	-	80	27	41	-	-	32	52
**Nikolajsen et al (2004)**	2	PF	8–12 cm (2–4 cm)	204	30	71	-	18	87	54
**Loos et al (2008)**	2	PF	12–15 cm (2–3 cm)	674	35	-	-	4	-	64
**Shahin et al (2009)**	1	PF vs ML	-	340 (161 vs 164)	26	-	-	-	100	0
**Naki et al (2009)**	1	PF vs ML	-	180 (90 vs 90)	27	-	-	-	-	-
**Kainu et al (2010)**	2	PF	-	205	34	67	27	26	67	-
**Fatusic et al (2011)**	2	PF vs ML	-	145 (60 vs 85)	28	-	-	-	-	-
**Ghahiry et al (2012)**	1	PF vs ML	-	112 (60 vs 52)	26	-	-	-	-	-
**Eisenach et al (2008/2013)**	1	PF	-	391	31	129	11	43	56	-
**Kiyac Altinbas et al (2013)**	1	PF	-	110	29	-	-	-	-	-

**Table 2:** Data about quality of life and acute/chronic pain of eligible population.

**Author (Year)**	**Abdominal opening technique**	**Number of patients (PF vs ML)**	**QoL**	**Pain following surgery**					**Pain time**	
				**24–48 hours**	**1–3 weeks**	**4–8 weeks**	**3–6 months**	**1 year and beyond**	**VAS acute**	**VAS chronic**
**Hojberg et al (1998)**	PF	40	-	-	-	-	-	-	1.5	-
**Ferrari et al (2000)**	PF vs ML	158 (75 vs 83)	-	-	-	-	-	-	2.5 vs 2.8	-
**Ansaloni et al (2001)**	PF	80	-	-	-	-	-	-	4.9	-
**Nikolajsen et al (2004)**	PF	204	-	-	121	58	14	27	-	3.5
**Loos et al (2008)**	PF	674	-	-	-	-	-	-	-	2.9
**Shahin et al (2009)**	PF vs ML	340 (161 vs 164)	-	-	-	-	-	-	6.1 vs 4.5	2.8 vs 2.7
**Naki et al (2009)**	PF vs ML	180 (90 vs 90)	-	-	-	-	-	-	1.8 vs 2.1	-
**Kainu et al (2010)**	PF	205	-	-	84	75	27	42	-	3.6
**Fatusic et al (2011)**	PF vs ML	145 (60 vs 85)	Bodily pain (72.4 vs 56.7); Social functioning (71.5 vs 60.4); Vitality (61.7 vs 50.3)	-	-	-	-	-	4.3 vs 2.8	3.8 vs 2.5
**Ghahiry et al (2012)**	PF vs ML	112 (60 vs 52)	-	-	-	-	-	-	-	4 vs 2
**Eisenach et al (2008/2013)**	PF	391	-	391	-	36	17	6	4.6	2.2
**Kiyac Altinbas et al (2013)**	PF	110	-	-	-	-	-	-	2	-

**Table 3:** Data about incomplete manuscripts (lacking data about VAS values, reporting acute and/or chronic pain outcome indirectly evaluated by analgesia request, duration of subjective pain, women satisfaction) also considered in systematic review.

**Author (Year)**	**Number of patients (PF vs ML)**	**Study Highlights**
**Luijendijk et al (1997)**	243	Report 57 cases of post-surgical chronic pain due to entrapment or neuroma formation. 61 patients report numbness in scar region.
**Darj et al (1999)**	50	Comparison between Pfannensteil and Misgav-Ladach technique. VAS is not reported. Analgesic drugs require is reported and compared.
**Franchi et al (2002)**	310	The study report better condition for Misgav-ladach technique in term of chronic pain.
**Almeida et al (2004)**	116	The study analyze chronic pain after low abdominal surgery detecting CS as risk factor.
**Tosun et al (2006)**	150	3 cases of pain six months from surgery and 1 case of persistent pain.
**Malvasi et al (2007)**	477	Only analgesic drugs require is reported and no difference between two techniques are reported.
**Nabhan et al (2008)**	600	Patients underwent Pfannensteil and Misgav-Ladach techniques for CS reported a comparable long-term pain (4/65).
**Gedikbasi et al (2009)**	111	Only analgesic drugs require is reported and Misgav-Ladach technique shows reduced need.

**1**: prospective studies; **2**: observational and retrospective studies, **PF**: Pfannenstiel technique; **ML**: Misgav-Ladach technique; **Qol**: Quality of Life.

Data on 4771 patients was collected. The majority of the population considered, 3531 patients, underwent a Pfannestiel procedure while the Misgav-Ladach technique was performed on the remaining 1240 patients. An abdominal vertical incision was recorded in 45 cases, which were excluded from the study. Median age was 30 years (range 26–35 years).

Three studies considered previous CS as exclusion criteria [9,14,21]; the remaining did not. Among the latter, only four studies [8,20,22,24] reported data about previous CS rate with a 37.46% weighted mean value (range 32.68%–49.38%). Concerning previous abdominal surgery rate, only five studies [11,20,22–24] reported a weighted mean value of 25.14% (range 4.01%–42.71%). Data regarding pre-existing abdominal pain before CS were reported in only two studies with a percentage of 11% and 27% respectively [20,22].

Data on anesthesia during CS was reported only in six studies: 71.52% of women received spinal anesthesia and the remaining general anesthesia. [8,11,20,22,24,25]

Concerning percentage of emergency CS, we note a homogenous distribution of both techniques: 41% for Pfannenstiel and 48% for Misgav-Ladach. [8,21,23,24,25]

Information regarding skin incision length, was reported in only two studies both of which, however, described the Pfannenstiel approach and where therefore not comparable: reported length ranged between 12–15 cm in the former and 8–12 cm in the latter. [23,24]

Only 4 studies reported data on postoperative pain rate with values of 100% in class 1, 50% in class 2, 21.1% in class 3, 7% in class 4 and 9,3% in class 5. None of the Authors distinguished data on the basis of the surgical technique used. [19,20,22,24]

A comparison between the two techniques was possible only through VAS score values: data on acute pain was reported in 9 studies for the Pfannenstiel technique [8,9,11,12,14,19–21,25] with a weighted mean value of 4.02, and in 5 studies for the Misgav-Ladach technique [8,9,14,21,25] with a weighted mean value of 3.26. (table 1,2). Data regarding chronic pain was reported in 7 studies for the Pfannenstiel technique with a weighted mean value of 3 and in 5 studies for the Misgav-Ladach technique with a weighted mean value of 1.97. [9,10,19,24,25]

Regarding women satisfaction rate, only one study reported that Misgav-Ladach method might lead to a better short term QoL. [9]

Data collected from the remaining 8 studies, which did not consider VAS, was controversial: some studies showed that analgesia request was comparable between the two designated groups,[29] while others reported an increase in the analgesia request in the Pfannenstiel group. [27,28] Moreover, pain resulted globally increased in the Pfannenstiel approach [3,27,28] especially when chronic pain was considered. [24,26,30,31] All data is summarized in Tables 1–3.

## Discussion

In Italy, CS rate increased from a low 23% in 1985 to a record 45.4% in 2006,confirming Edward Cragin’s 1916 dictum “once a caesarean, always a caesarean”. [33,34]

Since nearly one in three women undergoes caesarean delivery, a comprehensive understanding of a woman’s experience and subsequent QoL after surgery is important.

The long history of CS may explain the availability of several variants in surgical technique commonly in use today. Due to its wide diffusion, CS techniques should be analysed in order to reduce postoperative pain, minimize morbidity and ensure the best possible outcome.

Few reviews, two of which were Cochrane manuscripts, compared maternal outcomes after Pfannenstiel and Misgav-Ladach techniques. All concluded in agreement stating that the Misgav-Ladach incision demonstrated several advantages, including reduced post-operative pain. [4,13,35,36]. Most studies, however, reported exclusively on short-term postoperative pain and analgesic use without providing any information regarding long term outcomes and patient satisfaction.

Poor quality studies, incomplete pain description, use of different pain evaluation scales, short-term follow up and lesser consideration of pain as primary outcome made a complete comparison between the two abdominal opening techniques difficult in terms of maternal pain (acute and chronic) and patient satisfaction. We adjusted several data sets, which were reported in different scales, according to a recent comparative analysis system created to perform pain analysis processing [15].

Almost all works asserted that acute postoperative pain and maternal post CS analgesia requirements were lower in the Misgav-Ladach rather than in the Pfannenstiel groups. While a few studies reported neutral data, none reported lower pain after a Pfannenstiel incision.

The etiology and mechanism of different pain intensity was strictly linked to the surgical approach used and the subsequent abdominal anatomical area implicated.[23,37] In comparison to the Misgav-Ladach, the Pfannenstiel skin incision is smaller, but in a lower abdominal sectionSince lower abdominal wall innervation proceeds in a latero-medial direction, Pfannenstiel incision often involves nerve pathways, causing a possible iatrogenic damage. Additionally, in the Pfannenstiel technique sharp incision is used to access the abdominal layers (skin, subcutaneous, fascia and peritoneum ones) while in Misgav-Ladach approach, the incision is made only in the midline and then extended laterally through blunt finger dissection in the cranio-caudal direction, perpendicularly to the direction of nerve spreading. The result is minimal or absent of nerve damage: the intrinsic nerve structure elasticity allows a moderate traction without anatomical damage.

Moreover, if during Pfannenstiel incision dissection is excessively extended laterally, both the ileo-hypogastric and the ileo-inguinal nerves may be harmed, potentially creating a neuroma. The subsequent nerve retraction or entrapment in constricting sutures could be responsible for local hypoesthesia, hyperesthesia and acute/chronic pain. Treatment strategies range from locally administered, repeated, short-acting anesthetic injections (e.g. lidocain) which provide immediate but limited pain relief, to neurectomy of the affected nerve. [37]

Although chronic postpartum pain is most likely chiefly associated with neural defects incurred, the increased rate of postoperative bleeding, adhesions and fibrosis of the anterior abdominal wall associated with Pfannenstiel technique may contribute to increase the long term abdominal pain.[31]Maternal QoL is not considered as an outcome in English literature, with the exception of the work published by Fatusic et al.[9] In this study, the Misgav Ladach method seems to lead to a better short-time QoL, reducing postoperative complications, when compared to the Pfannenstiel method.

A better short-term health-related QoL is commonly acknowledged in women following birth by vaginal delivery as opposed to delivery by CS, independently from urgency of the performance. [38,39]

To the aim of increasing the rate of vaginal deliveries, the evaluation of pathological obstetric conditions able to interfere with vaginal delivery or known risk factors responsible for dystocia, the increasing use of analgesia during labor and the favoring of the patients’ choice to assume alternative positions during labor and delivery have been encouraged in a modern obstetrical care setting. [40–43] Considering the fact that in women undergoing vaginal delivery, emergent CS and elective CS, physical health-related QoL scores rather than mental health-related QoL scores showed strong differences between the categories [44].

The Joel-Cohen abdominal access may avoid the discomfort related to acute or chronic paresthesia/hypoesthesia, which is one of the co-factors impairing QoL after Caesaean delivery [9].

Pain reduction after CS, similarly to the use of epidural analgesia during labor, may likely influence social functioning and puerperal vitality by reducing the duration of post-partum hospitalization and consequent health costs in addition to improving maternal-fetal bonding and neonatal feeding [45].

To our knowledge, our study represents the first review comparing all available data regarding the two most frequently performed CS abdominal access techniques in terms of post-surgical acute/chronic pain and maternal QoL.

The main limitation is related to the difficulty in comparing data deriving from different tests (subjective or objective) which use different units of measure and end-points. Furthermore, many studies did not consider pain as the primary outcome and hardly any did pay attention to QoL. Long term outcomes were often poorly reported and the description of surgical approaches proved fragmentary. Moreover, few studies reported detailed data concerning status of previous CS or other abdominal surgery, history of abdominal/pelvic pain, CS type (elective or urgent) and length of skin incision.

## Conclusions

CS is one of the most commonly performed major abdominal operations, often performed by Surgeons with different levels of skill. The identification of the best standardized abdominal opening technique capable of reducing postoperative discomfort and thus increase QoL is necessary. To this aim, further studies should be planned. It is essential to take into account nerve topography because pain, due to nerve damage, seems to represent the most relevant cause of maternal postsurgical discomfort.

Standardized and validated tests may facilitate the assessment of maternal satisfaction in short and long-term follow-up programs: a low maternal satisfaction may represent a limiting factor in a woman’s desire for future pregnancy.

When planning further studies, it is mandatory that the history of chronic pelvic pain, previous abdominal surgery, length of skin incision, and requested analgesia be investigated to minimize the influence of confounding factors.

As of today, the available studies which report on this topic suggest that the Misgav-Ladach approach may be considered the gold standard surgical technique capable of to reducing postsurgical acute and chronic pain and improving puerperal QoL.

## Supporting Information

S1 PRISMA Checklist (DOC)
